# Single-cell RNA sequencing identifies *Fgf23*-expressing osteocytes in response to 1,25-dihydroxyvitamin D_3_ treatment

**DOI:** 10.3389/fphys.2023.1102751

**Published:** 2023-01-27

**Authors:** Ayako Hanai, Ayako Kawabata, Kenta Nakajima, Kazuhiro Masuda, Itaru Urakawa, Masahiro Abe, Yuji Yamazaki, Seiji Fukumoto

**Affiliations:** ^1^ R&D Division, Kyowa Kirin Co., Ltd., Tokyo, Japan; ^2^ Department of Endocrinology, Metabolism and Hematology, Tokushima University Graduate School of Medical Sciences, Tokushima, Japan; ^3^ Department of Molecular Endocrinology, Fujii Memorial Institute of Medical Sciences, Institute of Advanced Medical Sciences, Tokushima University, Tokushima, Japan

**Keywords:** FGF23, osteocyte, single-cell RNA sequencing, femur, transcriptome, gene expression, 1,25-dihydroxyvitamin D_3_ (calcitriol)

## Abstract

Fibroblast growth factor 23 (FGF23), a hormone, mainly produced by osteocytes, regulates phosphate and vitamin D metabolism. By contrast, 1,25-dihydroxyvitamin D_3_, the active form of vitamin D, has been shown to enhance FGF23 production. While it is likely that osteocytes are heterogenous in terms of gene expression profiles, specific subpopulations of *Fgf23*-expressing osteocytes have not been identified. Single-cell RNA sequencing (scRNA-seq) technology can characterize the transcriptome of an individual cell. Recently, scRNA-seq has been used for bone tissue analysis. However, owing to technical difficulties associated with isolation of osteocytes, studies using scRNA-seq analysis to characterize FGF23-producing osteocytes are lacking. In this study, we characterized osteocytes secreting FGF23 from murine femurs in response to calcitriol (1,25-dihydroxyvitamin D_3_) using scRNA-seq. We first detected *Dmp1*, *Mepe*, and *Phex* expression in murine osteocytes by *in situ* hybridization and used these as marker genes of osteocytes. After decalcification, enzyme digestion, and removal of CD45^+^ cells, femoral bone cells were subjected to scRNA-seq. We identified cell clusters containing osteocytes using marker gene expression. While *Fgf23* expression was observed in some osteocytes isolated from femurs of calcitriol-injected mice, no *Fgf23* expression was detected in untreated mice. In addition, the expression of several genes which are known to be changed after 1,25-dihydroxyvitamin D_3_ treatment such as *Ccnd2*, *Fn1*, *Igfbp7*, *Pdgfa*, and *Timp1* was also affected by calcitriol treatment in *Fgf23*-expressing osteocytes, but not in those lacking *Fgf23* expression, even after calcitriol administration. Furthermore, box-and-whisker plots indicated that *Fgf23* expression was observed in osteocytes with higher expression levels of the *Fam20c*, *Dmp1*, and *Phex* genes, whose inactivating mutations have been shown to cause FGF23-related hypophosphatemic diseases. These results indicate that osteocytes are heterogeneous with respect to their responsiveness to 1,25-dihydroxyvitamin D_3_, and sensitivity to 1,25-dihydroxyvitamin D_3_ is one of the characteristics of osteocytes with *Fgf23* expression. It is likely that there is a subpopulation of osteocytes expressing several genes, including *Fgf23*, involved in phosphate metabolism.

## Introduction

*FGF23* has been identified as a gene responsible for autosomal dominant hypophosphatemic rickets (ADHR Consortium, 2000). Fibroblast growth factor 23 (FGF23) has also been identified as a factor responsible for tumor-induced osteomalacia, a rare paraneoplastic syndrome characterized by hypophosphatemia (Shimada et al., 2001). FGF23 has been shown to be physiologically produced mainly by osteocytes (Liu et al., 2003) and to act as a hormone regulating phosphate and vitamin D metabolism (Shimada et al., 2004a; Shimada et al., 2004b). In addition to autosomal dominant hypophosphatemic rickets and tumor-induced osteomalacia, several types of hypophosphatemic rickets/osteomalacia have been shown to be caused by overexpression of FGF23 (Fukumoto and Martin, 2009). For example, X-linked hypophosphatemic rickets is caused by inactivating mutations in the *PHEX* gene located on the X chromosome, which has homology to a family of endopeptidases (Francis et al., 1995). It is known that the *Phex/PHEX* is expressed in mouse bones and in human bones, lungs, and ovaries (Du et al., 1996; Beck et al., 1997; Grieff et al., 1997; Guo and Quarles., 1997; Lipman et al., 1998). Autosomal recessive hypophosphatemic rickets 1 is caused by mutations in the *DMP1* gene (Feng et al., 2006; Lorenz-Depiereux et al., 2006). DMP1 is a glycoprotein that is highly expressed in the bones and teeth and controls mineralization (George et al., 1993). Raine syndrome is caused by mutations in the *FAM20C* gene (Simpson et al., 2007). *FAM20C* mutations were identified during whole-exome sequencing in patients with FGF23-related hypophosphatemia, dental anomalies, and ectopic calcification (Rafaelsen et al., 2013). In the absence of functional *PHEX*, *DMP1*, or *FAM20C*, *FGF23* expression is considered to be elevated in osteocytes and in circulation, leading to phosphate excretion from the kidneys and reduction in circulating phosphate (Martin et al., 2011; Rafaelsen et al., 2013; Miyagawa et al., 2014).

Recently, single-cell RNA sequencing (scRNA-seq) technology has been developed to characterize the transcriptomes of individual cells (Macosko et al., 2015; Zheng et al., 2017; Zilionis et al., 2017). This method has been used to map the complex cell diversity (Baryawno et al., 2019; Tikhonova et al., 2019) and identify gene expression signatures related to cell differentiation (Kanton et al., 2019; Wolock et al., 2019). Bone tissue is composed of various cells, and recently several scRNA-seq analyses data of bone cells have been reported (Ayturk et al., 2020; Wang et al., 2021). However, technical difficulties associated with isolation of osteocytes limit scRNA-seq analysis. Additionally, since *Fgf23* is normally expressed at considerably low levels in osteocytes (Liu et al., 2006), difficulties in detection of *Fgf23* gene expression are expected. Recently, Wang et al. performed scRNA-seq using murine femoral cells and demonstrated defects in osteocyte maturation in the absence of *Sp7*. They were able to isolate osteocytes using expression of *Dmp1* or *Mepe*, which encodes a bone matrix protein. However, in this study, *Fgf23*-expressing osteocytes were not detected (Wang et al., 2021).

Several studies have elucidated various signals that regulate FGF23 secretion. A high phosphate diet has been shown to increase FGF23 levels in both mice and humans (Ferrari et al., 2005; Perwad et al., 2005; Antoniucci et al., 2006). In addition to phosphate, 1,25-dihydroxyvitamin D_3_ has been reported to increase serum FGF23 levels (Saito et al., 2005). Moreover, *vitro* analysis using MC3T3-E1 osteocyte progenitor cells, 1,25-dihydroxyvitamin D_3_ induced FGF23 production, and *Fgf23* expression in osteocyte-like cells transfected with a knockdown sequence against vitamin D receptor was significantly decreased compared with that in control cells (Yashiro et al., 2020). In this study, we validated the utility of scRNA-seq to quantify the abundance and describe the characteristics of subpopulations of osteocytes from murine femurs. In particular, we focused on the characterization of *Fgf23*-expressing osteocytes responded to 1,25-dihydroxyvitamin D_3_.

## Materials and methods

### Animals and calcitriol injection

Twelve 7-week-old male C57BL/6J mice (The Jackson Laboratory Japan, Inc.) were divided into two groups: untreated and treated with calcitriol (Kyowa Kirin Co., Ltd.). The administration protocol was modified from previous reports (Kolek et al., 2005; Saito et al., 2005). Mice in treated group were intravenously injected with 2.5 μg/kg calcitriol every day for 3 days. Twenty-four hours after the final injection, the mice were euthanized under anesthesia, and femurs were collected for scRNA-seq analysis.

### Ethics approval statement

All animal studies were performed in accordance with the Standards for Proper Conduct of Animal Experiments at Kyowa Kirin Co., Ltd., under the approval of the company’s Institutional Animal Care and Use Committee. Tokyo Research Park of Kyowa Kirin Co., Ltd. is fully accredited by AAALAC International.

### *In situ* hybridization

DNA fragments corresponding to murine *Dmp1* cDNA (nucleotides 1129–1744), *Mepe* cDNA (nucleotides 810–1468), and *Phex* cDNA (nucleotides 239–1161) were individually cloned into pGEM-T vector (Promega K.K.). Sense and antisense probes were labeled with digoxigenin (DIG) using *in vitro* transcription. Paraffin-embedded blocks of mouse femurs were purchased from Genostaff Co., Ltd. Blocks were cut into 6 μm-thick sections and fixed on glass slides. The sections were acetylated with 0.2% hydrochloric acid and permeabilized with 4 μg/mL proteinase K (FUJIFILM Wako Pure Chemical Corporation) at 37°C for 10 min, with each step followed by two 5 min washes in PBS. Hybridization with DIG-labeled riboprobes was performed at 60°C for 16 h. For mRNA detection, serial sections were used for hybridization with sense and antisense riboprobes. The sections were then washed with 50% formamide in 0.5x G-Wash (Genostaff Co., Ltd.) and blocked with G-Block (Genostaff Co., Ltd.). The hybridized DIG-labeled RNA probes were detected by alkaline phosphatase–conjugated anti-DIG antibody (Roche Diagnostics) in G-Block-TBST and nitro blue tetrazolium chloride/5-bromo-4-chloro-3-indolyl phosphate (NBT/BCIP) substrates (Sigma-Aldrich). The sections were counterstained with nuclear fast red dye (Muto Pure Chemicals Co., Ltd.), fixed with G-Mount (Genostaff Co., Ltd.), cleared in xylene, and mounted using Marinol (Muto Pure Chemicals Co., Ltd.). Samples were imaged using a virtual slide scanner (NanoZoomer S210; Hamamatsu Photonics K.K.) at ×40 magnification. The collected images were adjusted for brightness.

### Femoral cell isolation

The femurs were pooled for each group and treated as one sample. Cells were isolated from mouse femurs utilizing a modified protocol derived from previous reports (Stern et al., 2012; Miyagawa et al., 2014). Briefly, epiphyses were cut off, and the marrow was flushed out by centrifugation. Mouse femurs were disrupted by Cryo-Press (Microtec Co., Ltd.) and digested with 1.6 U/mL collagenase D (Roche Diagnostics) in HBSS; pH = 7.8; FUJIFILM Wako Pure Chemical Corporation) for 30 min. The supernatant was discarded. Enzyme treatment and removal of supernatant steps were repeated twice. Residual bone pieces were treated with 20 mM ethylene glycol bis(β-aminoethylether)-N,N,N′,N′-tetraacetic acid disodium salt solution (neutral) (EGTA; Nacalai Tesque, Inc.) in HBSS for 15 min and then with 1.6 U/mL collagenase D for 20 min to collect the cells. Treatment with collagenase D was repeated twice. All digestion steps were carried out in a 3.5 mL solution in a six-well Petri dish, on a rotating shaker at 120 rpm in a 37°C incubator. After centrifugation and removal of supernatant, cell pellets were suspended in 1% (w/v) BSA-PBS(−); pH = 7.0 (Nacalai Tesque, Inc.). To remove CD45-positive cells, cell suspensions were mixed with CD45 microbeads (Miltenyi Biotec) and incubated at 4°C for 15 min in PEB buffer and rinsed. PEB buffer contains MACS^®^ BSA stock solution (Miltenyi Biotec) and autoMACS rinsing solution (Miltenyi Biotec) in a 1:20 ratio. Cell suspensions were passed through the LD column (Miltenyi Biotec). The flow-through was collected, centrifuged, and resuspended in 1% (w/v) BSA-PBS(−). Cells were passed through a 40 μm nylon strainer and used for scRNA-seq.

### scRNA-seq library preparation and sequencing

For each sample, the quantity and viability of the cells were evaluated. Absence of aggregated cells or cell debris was confirmed microscopically. Single cells were encapsulated into emulsion droplets using Chromium Connect (10x Genomics). scRNA-seq libraries were constructed using Chromium Next GEM Automated Single Cell 3′ Library and Gel Bead Kit v. 3.1 (10x Genomics), Chromium Next GEM Automated Chip G Single Cell Kit, and Single Index Kit T Set A (10x Genomics) according to the manufacturer’s protocol. The libraries were sequenced using Illumina NextSeq 2000.

### Data processing for scRNA-seq datasets

Sequencing reads (FASTQ files) were mapped to the mouse reference genome (mm10) using Cell Ranger (v. 4.0.0; 10x Genomics; https://support.10xgenomics.com/single-cell-gene-expression/software/overview/welcome). The downstream analysis was performed using the Seurat R package (version 3.2.2). The output matrices from Cell Ranger were converted into Seurat objects and barcodes with <200 genes were excluded to eliminate low-quality cells or cell-free droplets as a primary quality control. Following the primary quality control, each object was normalized and scaled according to the Seurat standard workflow, followed by principal component analysis based on the top 2000 highly variable genes, Uniform Manifold Approximation and Projection (UMAP) dimension reduction, and unsupervised clustering. After the clusters were obtained, the clusters with a high mitochondrial gene fraction (median of percent. mt >10%), low gene counts (median of nFeature <1000), and low Unique Molecular Identifier (UMI) counts (median of nCount <2000) were excluded to eliminate dead cells as the secondary quality control. Following the secondary quality control, the objects were reprocessed following the Seurat standard workflow and subsequently integrated by the Seurat anchor-based integration workflow followed by principal component analysis, dimensionality reduction, and unsupervised clustering. The UMAP coordinate and the unsupervised cluster information of each cell was incorporated in the cloupe file for downstream analysis.

### Real-time quantitative PCR

Total RNA was isolated with a phenol-chloroform extraction method using ISOGEN kit (Nippon Gene Co., Ltd.) according to the manufacturer’s protocol, and reverse transcribed using QuantiTect^®^ Reverse Transcription Kit (QIAGEN). cDNA from 1 µg of total RNA was subjected to real-time PCR. TaqMan Gene Expression Master Mix (Life Technologies Co., Ltd.) was used for real-time quantitative PCR. The primer sets were obtained from Applied Biosystems (*Fgf23*, no. Mm00445621_m1; *Actb*, no. Mm02619580_g1). Reactions were run on and analyzed with a QuantStudio™ 5 real-time PCR system (Thermo Fisher Scientific K.K.). *Fgf23* mRNA expression levels were normalized with respect to those of *Actb* mRNA.

### Serum FGF23 measurements

Serum FGF23 levels were measured in mice using a sandwich enzyme-linked immunosorbent assay kit (Kainos Laboratories) (Yamazaki et al., 2002).

## Results

### Detection of *Dmp1*, *Mepe*, and *Phex* gene expression by *in situ* hybridization

**FIGURE 1:**
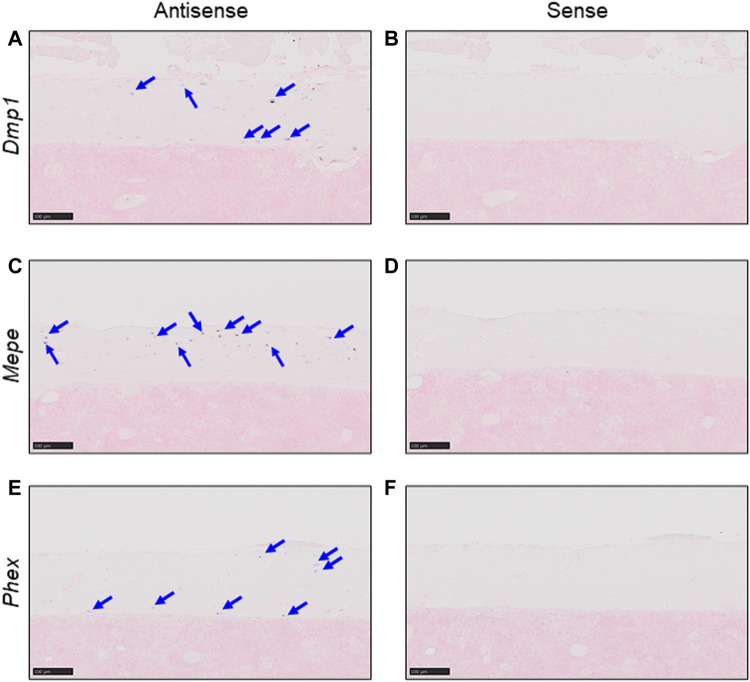
Detection of osteocyte marker mRNA using *in situ* hybridization. Expression of osteocyte markers was detected in serial sections from untreated C57BL/6J murine femurs by *in situ* hybridization. **(A,B)** Expression of *Dmp1* in cortical bone. **(C,D)** Expression of *Mepe* in cortical bone. **(E,F)** Expression of *Phex* in cortical bone. Markers detected using antisense probes [panels **(A, C, E)**] and sense probes [panels **(B, D, F)**] (Scale bar: 100 μm).

We first confirmed the expression of *Dmp1*, *Mepe*, and *Phex*, genes which are already known to be expressed in osteocytes, by *in situ* hybridization of murine femur cortical bone tissue (Petersen et al., 2000; Gluhak-Heinrich et al., 2003; Kalajzic et al., 2004; Lu et al., 2004; Dallas et al., 2013). Positive signals were detected only with antisense probes for each gene (Figures 1A, C, E), and no non-specific signals were detected with sense probes (Figures 1B, D, F). Thus, in this scRNA-seq analysis, we defined a cell cluster with higher expression levels of *Dmp1*, *Mepe*, and *Phex* as osteocytes.

### scRNA-seq identifies diverse murine femoral cell populations and their transcriptomes

**FIGURE 2:**
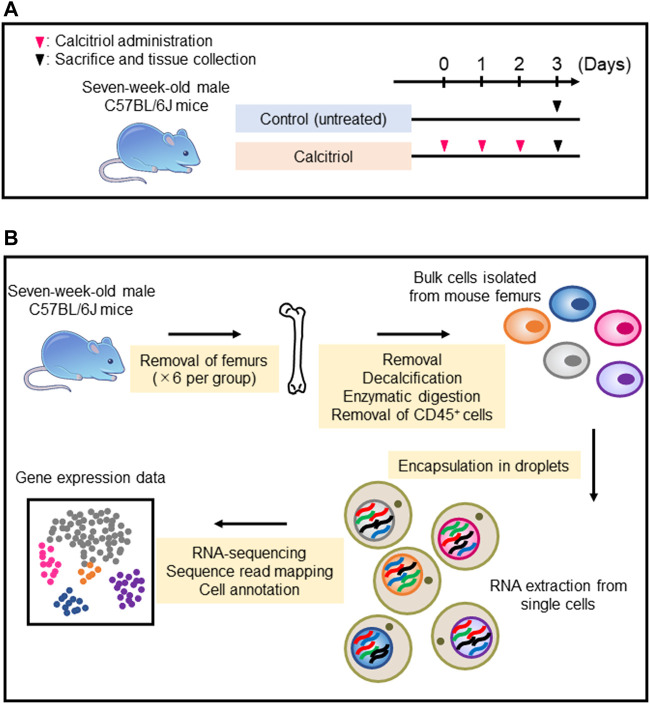
Single-cell RNA sequencing sample preparation and RNA sequencing protocol. **(A)** Seven-week-old male C57BL/6J mice were injected with 2.5 μg per kg of calcitriol (*n* = 6) every day for 3 days. As a control, six untreated C57BL/6J mice were sacrificed on day 3. **(B)** Each cell isolated from six femurs by collagenase digestion and EGTA treatment was encapsulated in a droplet for mRNA extraction and RNA sequencing.

C57BL/6J mice were injected with calcitriol every day for 3 days. Untreated C57BL/6J mice were used as controls (Figure 2A). The femurs from six mice per group were collected, and used for isolation of femoral cells. Subsequently, scRNA-seq was performed on isolated cells (Figure 2B). More than 60,000 cells could be collected with a cell viability rate of over 80% from each group sample (Table 1), which were used to generate scRNA-seq libraries. We obtained over 300 million reads from the femoral single-cell libraries in each group. In the control group, 51,839 reads per cell and 6,076 cells per library were obtained. On the other hand, 69,689 reads per cell and 4,562 cells per library were obtained in the calcitriol-injected group. These libraries were used for preprocessing and filtering (Table 2).

**TABLE 1:** Information of isolated cells.

Group	Number of cells	Cell viability (%)
Untreated (Control)	1.66 × 10^5^	86
Calcitriol treatment	0.64 × 10^5^	96

**TABLE 2:** Results of single-cell RNA sequencing analyses.

Group	Number of reads	Estimated number of cells	Mean reads per cell	The median number of genes detected per cell-associated barcode
Untreated (Control)	314,975,207	6,076	51,839	808
Calcitriol treatment	317,920,929	4,562	69,689	2,449

**FIGURE 3:**
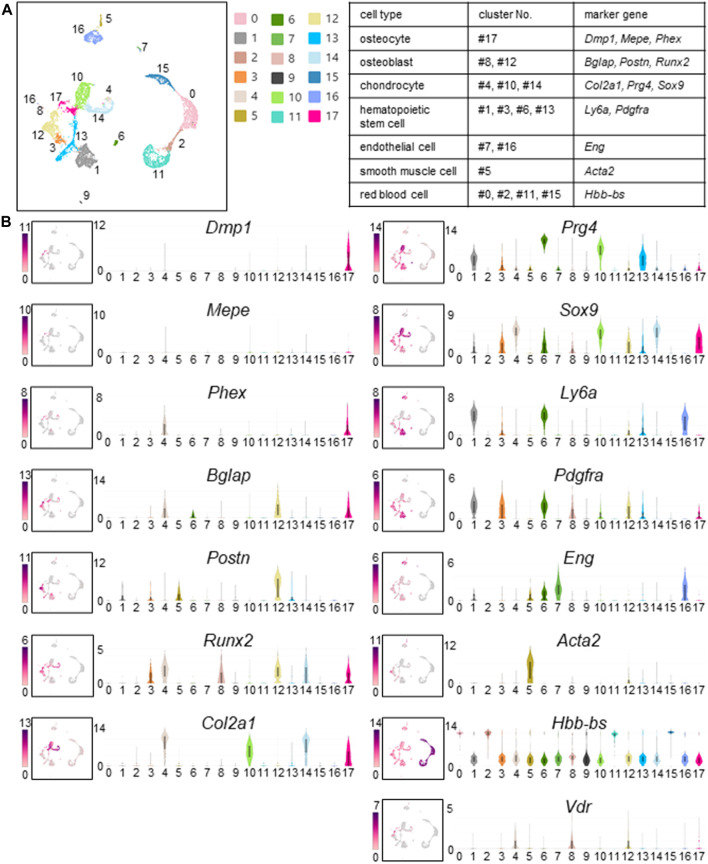
Cell clusters identified in fresh cells isolated from murine femurs. **(A)** Cell clustering was performed on cells isolated from femurs of calcitriol-injected and untreated C57BL/6J mice. Uniform Manifold Approximation and Projection (UMAP) plot wherein each dot represents a single cell, and cells sharing the same color code indicate discrete populations of transcriptionally similar cells. Table shows the correspondence between cell type, cluster number, and marker gene when identified. **(B)** UMAP plots of gene expression (left) and associated violin plots (right) indicate cluster-specific expression of representative genes. Osteocytes are marked by expression of *Dmp1*, *Mepe*, and *Phex* (#17). Similarly, osteoblasts express *Bglap*, *Postn*, and *Runx2* (#8 and #12). Chondrocytes express *Col2a1*, *Prg4*, and *Sox9* (#4, #10, and #14). Hematopoietic stem cells express *Ly6a* and *Pdgfra* (#1, #3, #6, and #13). Endothelial cells express *Eng* (#7 and #16). Smooth muscle cells are distinctly marked by the expression of *Acta2* (#5). Red blood cells express *Hbb-Bs* (#0, #2, #11, and #15). *Vdr* expression that dictates calcitriol responsiveness is shown by UMAP plot and violin plot. *Vdr* was expressed in osteoblasts, chondrocytes and osteocytes (#4, #8, #12, and #17).

Murine femoral cells were divided into 18 clusters using Seurat’s unbiased cluster detection algorithms (Figure 3A). Cluster #17 was identified as an osteocyte cluster based on expression of *Dmp1*, *Mepe*, and *Phex*, which had been defined as osteocyte markers (Figure 3B). In other cell types, clusters were identified based on the expression profiles of osteoblast, chondrocyte, hematopoietic stem cell, endothelial cell, smooth muscle cell, and red blood cell marker genes that have been reported previously (Spangrude and Brooks., 1993; Ducy et al., 1996; Komori et al., 1997; Letamendía et al., 1998; Bi et al., 1999; Horiuchi et al., 1999; Ikegawa et al., 2000; Wang et al., 2006; Runck et al., 2009; Toffalini and Demoulin, 2010). Clusters #8 and #12 exhibited osteoblast markers (e.g., *Bglap*, *Postn*, and *Runx2*). Clusters #4, #10, and #14 exhibited chondrocyte markers (e.g., *Col2a1*, *Prg4*, and *Sox9*). The remaining femoral cell clusters expressed transcripts found in hematopoietic stem cells (*Ly6a* and *Pdgfra*; clusters #1, #3, #6, and #13), endothelial cells (*Eng*; clusters #7 and #16), smooth muscle cells (*Acta2*, cluster #5), and red blood cells (*Hbb-bs*; clusters #0, #2, #11, and #15) (Figure 3B). *Vdr* expression that dictates calcitriol responsiveness was detected in clusters #4, #8, #12, and #17, that contain chondrocytes (#4), osteoblasts (#8, #12), and osteocytes (#17). (Figure 3B).

### Detection of *Fgf23*-expressing osteocytes in femurs of calcitriol-injected mice

**FIGURE 4:**
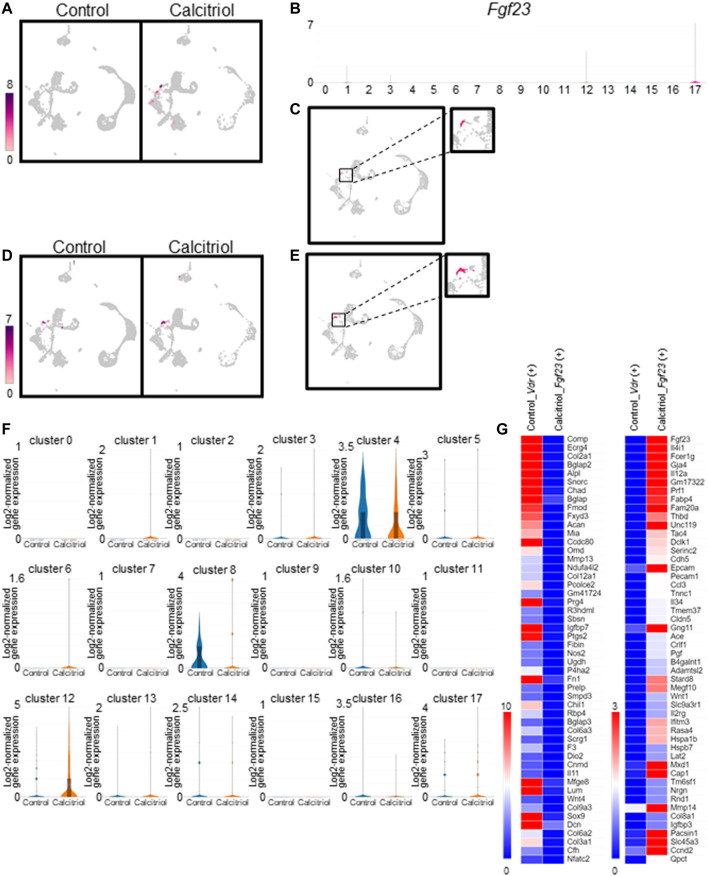
Identification and characterization of *Fgf23*-expressing cells in fresh cells isolated from femurs of calcitriol-injected mice. **(A)** Uniform Manifold Approximation and Projection (UMAP) plot showing *Fgf23* gene expression in all cells from calcitriol-injected mice or control mice. Color scale bar shows log2-normalized gene expression values. **(B)** Violin plots indicate cluster-specific expression of *Fgf23* gene. **(C)** UMAP plot showing *Fgf23* gene expression in osteocytes (cluster #17) of calcitriol-injected mice. **(D)** UMAP plot showing *Sost* gene expression in all cells from calcitriol-injected mice or control mice. Color scale bar shows log2-normalized gene expression values. **(E)** UMAP plot showing *Sost* gene expression in osteocytes (cluster #17) of calcitriol-injected mice. **(F)** Violin plots indicate *Vdr* gene expression in the untreated and calcitriol-injected mice in each cluster. **(G)** The gene expression was compared between *Fgf23*-expressing osteocytes in calcitriol-injected group and *Vdr*-expressing osteocytes in control group. Heat maps indicate gene expression profiles upregulated (right) or downregulated (left) in *Fgf23*-expressing osteocytes in calcitriol-injected group.

We compared the number of *Fgf23*-expressing cells between untreated and calcitriol-injected murine femoral cells (Figure 4A). *Fgf23*-expressing cells were not detected in the untreated mice. On the other hand, *Fgf23-*expressing cells were observed in clusters #1, #3, #12, and #17 of calcitriol-injected murine femoral cells. Eight *Fgf23-*expressing cells were observed in cluster #17 (osteocytes) of calcitriol-injected murine femoral cells (Figures 4A, B). In the calcitriol-injected group, *Fgf23* mRNA levels in cells isolated from femurs were higher than in the control group as measured by real-time quantitative PCR (Supplementary Figure S1). We compared another osteocyte marker gene, *Sost*, between untreated and calcitriol-injected murine femoral cells. *Sost*-expressing cells were detected at the same existence in both of the two groups (Figures 4D, E). Matching the distribution of *Fgf23* expression with the cell type of the cluster revealed that some of the osteocytes classified in cluster #17 expressed *Fgf23* (Figure 4C). Serum FGF23 levels were also higher in the calcitriol-injected group compared with the control group (Supplementary Figure S2).

To estimate the origin of *Fgf23*-expressing cells, we compared gene expression profiles in *Vdr*-expressing cells in the control group and *Fgf23*-expressing cells in the calcitriol-injected group. *Vdr*-expressing cells were observed in clusters #4, #8, #12, and #17 (Figure 3B). In clusters #4 and #8, *Vdr* gene expression was higher in the control group than in calcitriol-injected group (Figure 4F). This suggested that the percentage of *Vdr*-high expressing cells in cluster #4 and #8, i.e., chondrocytes and osteoblasts, was reduced by calcitriol treatment. We compared the gene expression profiles in *Vdr*-expressing osteocytes (cluster #17) in the control group and *Fgf23*-expressing osteocytes in the calcitriol-injected group. As a result, osteoblast and chondrocyte markers such as *Bglap*, *Col2a1*, *Prg4*, and *Sox9* (Ducy et al., 1996; Bi et al., 1999; Ikegawa et al., 2000) were detected as downregulated genes, especially in *Fgf23*-expressing cells (Figure 4G).

### *Fgf23*-expressing osteocytes are defined by specific gene expression profiles

**FIGURE 5:**
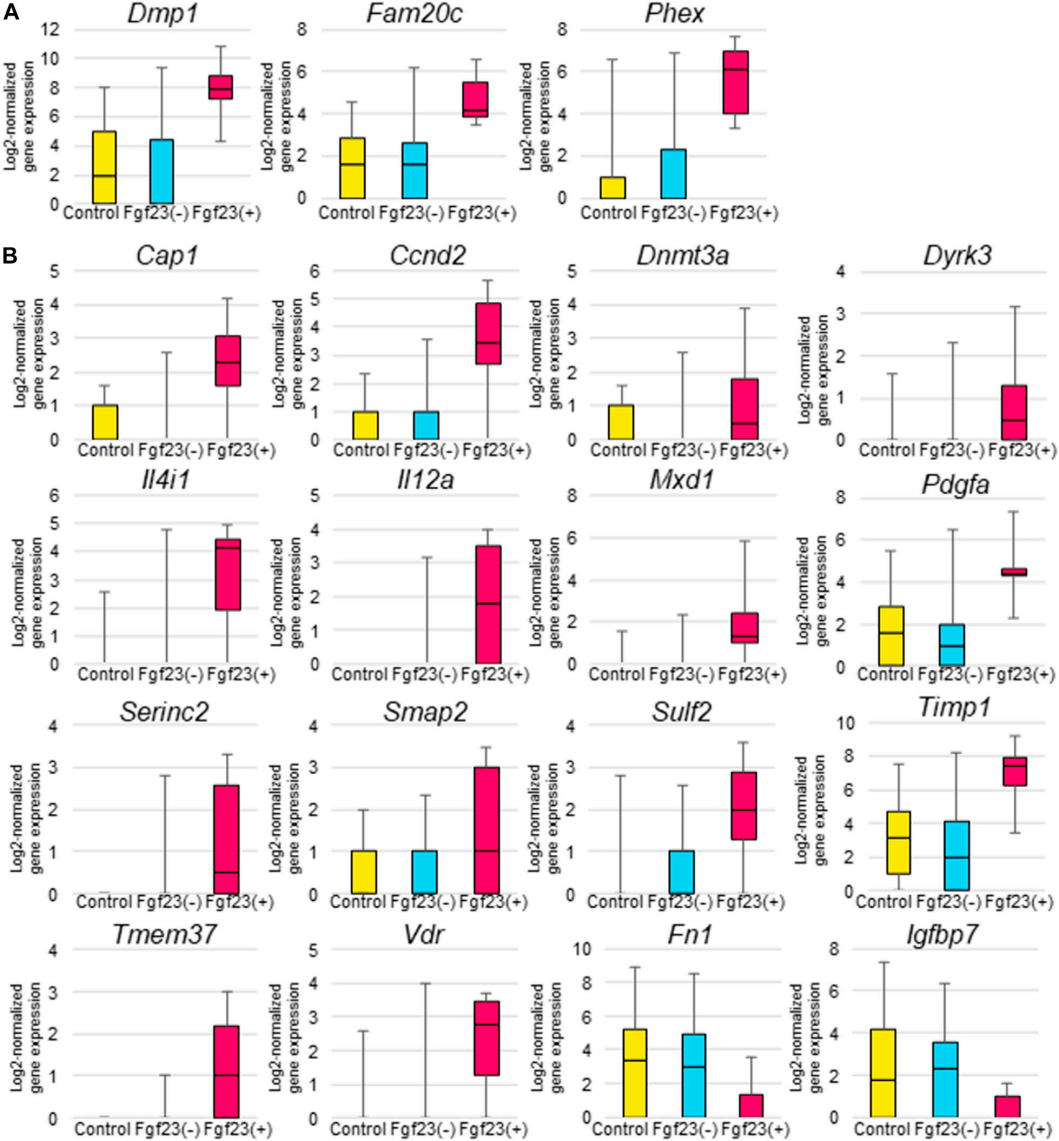
Comparison of gene expression levels among *Fgf23*-expressing osteocytes and non-*Fgf23*-expressing osteocytes from femurs of untreated or calcitriol-injected mice. **(A)** Box-and-whisker plots indicate *Dmp1*, *Fam20c*, and *Phex* gene expression in osteocytes from untreated mice, non-*Fgf23*-expressing osteocytes from calcitriol-injected mice, and *Fgf23*-expressing osteocytes from calcitriol-injected mice. The Y-axes of the plots show log2-normalized gene expression values. **(B)** Box-and-whisker plots indicate 1,25-dihydroxyvitamin D_3_–stimulated gene expression in untreated osteocytes from untreated mice, non-*Fgf23*-expressing osteocytes from calcitriol-injected mice, and *Fgf23*-expressing osteocytes from calcitriol-injected mice. The vertical axes of the plots show log2-normalized gene expression values.

To characterize *Fgf23*-expressing osteocytes, we compared the expression levels of *Dmp1*, *Fam20c*, and *Phex* genes, whose inactivating mutations have been shown to cause FGF23-related hypophosphatemic diseases, between osteocytes isolated from the control mice, as well as non–*Fgf23*-expressing osteocytes and *Fgf23*-expressing osteocytes isolated from the calcitriol-treated mice. *Fgf23*-expressing osteocytes showed higher expression levels of *Dmp1*, *Fam20c*, and *Phex* genes (Figure 5A). In the calcitriol-injected group, *Dmp1* mRNA levels in cells isolated from femurs were higher than in the control group as measured by real-time quantitative PCR (Supplementary Figure S3). We further compared gene expression profiles in these osteocytes. The expression levels of several genes that are known to be enhanced by 1,25-dihydroxyvitamin D_3_, such as *Cap1*, *Ccnd2*, *Dnmt3a*, *Dyrk3*, *Il12a*, *Il4i1*, *Mxd1*, *Pdgfa*, *Serinc2*, *Sulf2*, *Timp1*, *Tmem37*, and *Vdr,* were increased by calcitriol in *Fgf23*-expressing osteocytes compared with untreated osteocytes (Zhang et al., 1995; Timms et al., 2002; Satoh et al., 2005; Wang et al., 2005; An et al., 2010; Ramagopalan et al., 2010; Heikkinen et al., 2011; Salehi-Tabar et al., 2012; Ding et al., 2013; Goeman et al., 2014; Ryynänen et al., 2014; Seuter et al., 2014; Salehi-Tabar et al., 2019; Fernández-Barral et al., 2020). The expression of *Igfbp7* and *Fn1* genes, which are also known to be suppressed by 1,25-dihydroxyvitamin D_3_, was decreased by calcitriol in *Fgf23*-expressing osteocytes (Shang et al., 2000; Zhang et al., 2008; Satoh and Tabunoki., 2013) (Figure 5B). The expression levels of these genes were not influenced in osteocytes lacking *Fgf23* expression even after calcitriol injection (Figure 5B).

## Discussion

Osteocytes are embedded in the mineralized bone matrix, connecting and interacting with each other *via* gap junctions and through a bone fluid flow (Parfitt, 1977; Kamioka et al., 2001). One of the distinguishing characteristics of osteocytes is their lifespan, which can extend up to several decades in the bone matrix (Lanyon, 1993). However, recent studies have demonstrated the role of osteocytes as secretory cells as well (Bonewald, 2011). Osteocytes can organize bone remodeling, control calcium and phosphate homeostasis, and transmit signals to distant tissues. Recent studies also show that they have high heterogeneity in gene expression. For example, matured and deeply embedded osteocytes express high level of sclerostin, which is the product of the *SOST* gene (Dallas et al., 2013).

In this study, we focused on the genetic definition of *Fgf23*-expressing osteocytes. FGF23 is already known to be expressed in bone cells, especially in osteocytes (Feng et al., 2006; Liu et al., 2006). In addition to FGF23, we know that some proteins specifically expressed in osteocytes play critical roles in phosphate homeostasis, for example, PHEX and DMP1 (Bonewald, 2007; Bonewald and Johnson., 2008). *Dmp1*, *Mepe*, and *Phex* are highly expressed in osteocytes compared with osteoblasts or other cell types (Petersen et al., 2000; Gluhak-Heinrich et al., 2003; Kalajzic et al., 2004; Lu et al., 2004; Dallas et al., 2013). We were able to detect *Dmp1*, *Mepe*, and *Phex* expression specifically in osteocytes using *in situ* hybridization (Figure 1). By using these genes as marker genes, we could define osteocytes in scRNA-seq analysis (Figure 3).

To characterize femoral cells including osteocytes, we used the scRNA-seq technology. Ayturk et al. performed scRNA-seq using neonatal mouse calvarial cells and compared the relative cell type abundance and the transcriptomes of freshly isolated cells. However, in this study, osteocytes were not detected from neonatal murine calvarial cells (Ayturk et al., 2020). This is probably because osteocytes are present in the bone matrix and it is difficult to isolate them. In our current study, we focused on osteocyte detection from murine femurs. We optimized the protocols for tissue disruption, enzyme digestion, and depletion of CD45^+^ cells by MACS protocol (Figure 2), and we were able to detect osteocytes as a cell cluster that was defined by osteocyte-specific marker gene expression (Figure 3A). In addition, we defined other cell types using marker genes for osteoblasts, chondrocytes, hematopoietic stem cells, endothelial cells, smooth muscle cells, and red blood cells (Figure 3B). We obtained transcriptome profiles of murine femurs and analyzed characteristics of each cell type (Figure 3).

FGF23 is normally expressed at considerably low levels in osteocytes but its expression is considered to be increased in patients with hypophosphatemic rickets (Liu et al., 2006; Endo et al., 2008) and in patients with chronic kidney disease (Pereira et al., 2009). We could not detect *Fgf23*-expressing osteocytes by scRNA-seq from untreated murine femurs (Figure 4A). Our results are consistent with the previous reports.

To isolate *Fgf23*-expressing osteocytes, mice were injected with calcitriol. 1,25-dihydroxyvitamin D_3_ is known to induce expression of *Fgf23* in the osteocytes (Liu et al., 2006; Woo et al., 2011), suggesting a negative feedback system. The induction of *Fgf23* by 1,25-dihydroxyvitamin D_3_ is mediated by vitamin D receptor (Saji et al., 2010; Yashiro et al., 2020). In this study, *Vdr* gene expression was detected in osteoblasts, chondrocytes, and osteocytes (Figure 3A). It is known that *Vdr* is expressed in immature osteoblasts and chondrocytes (Wang et al., 2014). After calcitriol treatment, we could detect *Fgf23* expression in some cells that we had categorized as osteocytes (Figure 4A). Osteoblast and chondrocyte markers, *Bglap*, *Col2a1*, *Prg4*, and *Sox9* (Ducy et al., 1996; Bi et al., 1999; Ikegawa et al., 2000) were downregulated in *Fgf23*-expressing cells compared with *Vdr*-expressing cells in control group (Figure 4G). From these results, it was presumed that calcitriol treatment enhanced differentiation of *Vdr*-expressing osteoblasts into osteocytes, and increased the number of *Fgf23*-expressing cells. VDR is important in the late stage of osteogenic differentiation (Yang et al., 2018). Our results are consistent with the previous reports.

To genetically characterize *Fgf23*-expressing osteocytes, we analyzed the gene expressions regulating *Fgf23* expression. DMP1, PHEX, and FAM20C proteins are highly expressed in osteocytes and regulate FGF23 production and bone mineralization (Francis et al., 1995; Thompson et al., 2002; Bonewald, 2007; Kinoshita et al., 2014). In *Fgf23*-expressing osteocytes responding to calcitriol treatment, the expression of *Dmp1*, *Phex*, and *Fam20c* was higher than in non-*Fgf23*-expressing osteocytes (Figure 5A). However, the expression levels of these genes were variable in osteocytes from the untreated mice. It is probable that some osteocytes in untreated mice had expression levels of *Dmp1, Phex,* and *Fam20c* that were similar to those in *Fgf23-*expressing osteocytes. Since there have been no previous reports indicating that 1,25-dihydroxyvitamin D_3_ upregulates the expression of these genes, these results suggest that there is a population of osteocytes with higher expression levels of *Dmp1, Phex,* and *Fam20c* that can produce FGF23 in response to 1,25-dihydroxyvitamin D_3_.

Mutations in *PHEX*, *DMP1*, and *FAM20C* have been reported to be responsible for FGF23-related hypophosphatemia. Inactivating mutations in these genes cause the hypophosphatemic diseases X-linked hypophosphatemic rickets, autosomal recessive hypophosphatemic rickets 1, and Raine syndrome, respectively (George et al., 1993; Francis et al., 1995; Feng et al., 2006; Simpson et al., 2007). Therefore, it seems rather paradoxical that FGF23*-*producing osteocytes also express these genes. However, serial analysis of gene expression identified *DMP1* and *PHEX* as overexpressed genes in tumors responsible for tumor-induced osteomalacia and secreting FGF23 (De Beur et al., 2002). Additionally, *Dmp1* and *Fam20c* expression levels were higher in osteocytes from Hyp mice compared with those from wild-type mice (Miyagawa et al., 2014). While the detailed mechanism is unclear, our results suggest that PHEX, DMP1, and FAM20C are involved in the regulation of FGF23 production in a cell-autonomous manner.

There are several limitations in our study. Osteocytes make up 90%–95% of cells in the bone tissue (Bonewald, 2007), However, the number of isolated osteocytes and *Fgf23*-expressing osteocytes were small. Since isolation of single cells from bones is a time-consuming procedure, it is possible that the gene expression profiles of isolated cells are different from their profiles *in vivo*. Additionally, we could not detect *Fgf23* expression in bone cells in untreated mice. Further refinement of the methods for preparing bone cells would be necessary to address these issues.

In this study, we detail the first report of *Fgf23*-expressing osteocytes isolated using scRNA-seq. We also show that osteocytes are heterogeneous with respect to their responsiveness to 1,25-dihydroxyvitamin D_3_, and sensitivity to active vitamin D is one of the characteristics of osteocytes with *Fgf23* expression. It is likely that there is a subpopulation of osteocytes which expresses several genes, including *Fgf23*, that can affect phosphate metabolism.

## Data Availability

The datasets presented in this study can be found in online repositories. The names of the repository/repositories and accession number(s) can be found below: https://www.ncbi.nlm.nih.gov/search/all/?term=GSE220836.

## Abbreviations

cDNA, complementary DNA; DIG, digoxigenin; mRNA, messenger RNA; PCR, polymerase chain reaction; scRNA-seq, single-cell RNA sequencing.
