# Supplementary material for: Single-cell RNA sequencing identifies Fgf23-expressing osteocytes in response to 1,25-dihydroxyvitamin D3 treatment
Source: Front Physiol. 2023 Jan 27;14:1102751. doi: 10.3389/fphys.2023.1102751 (PMC9911654; doi:10.3389/fphys.2023.1102751)
Supplement: Supplementary file 1 [file DataSheet2.PDF]

Supplementary Material

Supplementary Figures

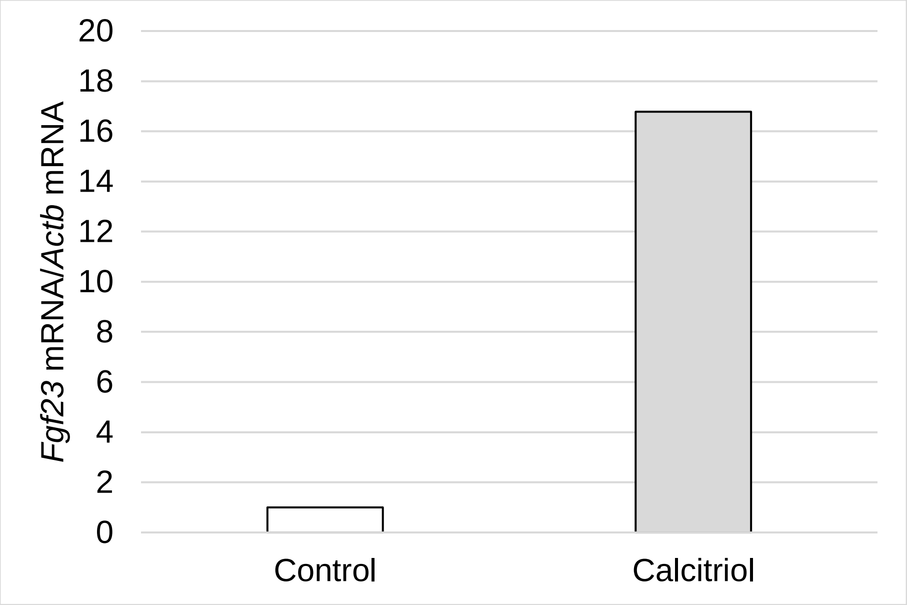

Supplementary Figure 1 Quantification of Fgf23 mRNA in isolated mouse femoral cells by real-time quantitative PCR. Expression of *Fgf23* was measured in isolated femoral cells from untreated (control) or calcitriol-treated C57BL/6J mice by real-time quantitative PCR. *Fgf23* expression levels were normalized by *Actb* gene expressions. Expression levels were expressed relative to the control group.

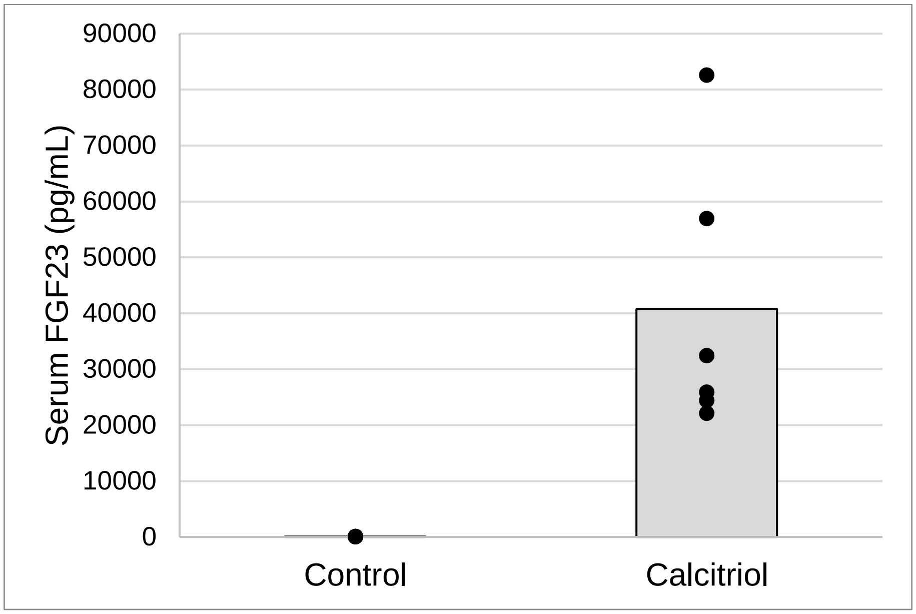

Supplementary Figure 2 Serum FGF23 levels in control and calcitriol-treated mice.
Concentrations of circulating FGF23 in untreated (control) mice or calcitriol-treated mice (24 h after the third injection of calcitriol) were analyzed using an FGF23 ELISA kit.
ELISA, Enzyme-linked immuno-sorbent assay

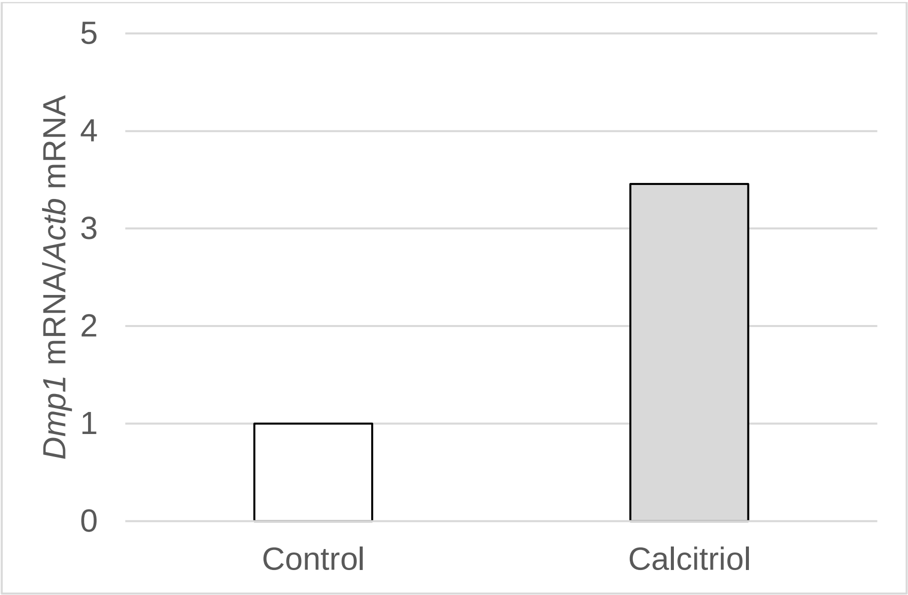

Supplementary Figure 3 Quantification of Dmp1 mRNA in isolated mouse femoral cells by real-time quantitative PCR. Expression of *Dmp1* was measured in isolated femoral cells from untreated (control) or calcitriol-treated C57BL/6J mice by real-time quantitative PCR. *Dmp1* expression levels were normalized by *Actb* gene expressions. Expression levels were expressed relative to the control group.
